# Supplementary material for: The impact of the strategic action plan to combat chronic non-communicable diseases on hospital admissions and deaths from cardiovascular diseases in Brazil
Source: PLoS One. 2022 Jun 8;17(6):e0269583. doi: 10.1371/journal.pone.0269583 (PMC9176809; doi:10.1371/journal.pone.0269583)
Supplement: S1 File — (PDF) [file pone.0269583.s001.pdf]

Complete Statistical Analysis.

Table1. Number of hospitalizations per 100.000 population per region and year

	GL	SQ	SQM	Value F	P-value
Region	4	1755828.61	438957.15	804.48	<0.001
Year	11	22564.74	2051.34	3.76	<0.001
Residual	44	24008.09	545.64		

ANOVA Two-way

We notice, through the p-value, that there is a difference for both Region (p-value= <0.001) as for Year (p-value= <0.001). The averages of Hospitalizations/100,000hab in orderregion were: North (average=319.53, SD=30.25), Northeast (average=439.75, SD=12.89), Midwest (average=517.58, SD=51.52), Southeast (average=587.52, SD=16.06), South (average=831.38, SD=15.5). The test of comparisons tukey multiples pointed out differences between all groups studied, such as we can to see at table a follow:

	Difference	IC.95	P.value
Northeast-Center West	-77.8	(-105, -50.7)	<0.001
North-Center West	-198.1	(-225.2,-170.9)	<0.001
Southeast-Center West	69.9	(42.8, 97.1)	<0.001
South-Center West	313.8	(286.7, 340.9)	<0.001
North, Northeast	-120.2	(-147.3, -93.1)	<0.001
Southeast-Northeast	147.8	(120.6, 174.9)	<0.001
south-northeast	391.6	(364.5, 418.8)	<0.001
Southeast-North	268.0	(240.9, 295.1)	<0.001
South North	511.9	(484.7, 539)	<0.001
south-southeast	243.9	(216.7, 271)	<0.001

The averages of Hospitalizations/100,000hab in ascending order of Year were: 2017 (average=514.19, SD=208.35), 2016 (average=514.27, SD=203.31), 2018 (average=519.66, SD=208.15), 2015 (average=522.18, SD=189.44), 2019 (average=527.31, SD=212.79), 2014 (average=534.8, SD=184.55), 2013 (average=541.14, SD=173.03), 2012 (average=543.37, SD=176.48), 2008 (average=556.19, SD=194.01), 2011 (average=561.26, DP=177.76), 2010 (average=565.78, DP=189.31), 2009 (average=569.71, DP=188.4). The
test in comparisons multiple in tukey pointed at following differences:

	**Difference**	**IC.95**	**P.value**
2009-2008	13.5	(-37.4, 64.5)	0.999
2010-2008	9.6	(-41.4, 60.5)	1,000
2011-2008	5.1	(-45.9, 56)	1,000
2012-2008	-12.8	(-63.8, 38.1)	0.999
2013-2008	-15.1	(-66, 35.9)	0.996
2014-2008	-21.4	(-72.3, 29.6)	0.947
2015-2008	-34.0	(-85, 16.9)	0.490
2016-2008	-41.9	(-92.9, 9)	0.200
2017-2008	-42.0	(-93, 9)	0.198

2018-2008	-36.5	(-87.5, 14.4)	0.383
2019-2008	-28.9	(-79.8, 22.1)	0.719
2010-2009	-3.9	(-54.9, 47)	1,000
2011-2009	-8.4	(-59.4, 42.5)	1,000
2012-2009	-26.3	(-77.3, 24.6)	0.818
2013-2009	-28.6	(-79.5, 22.4)	0.732
2014-2009	-34.9	(-85.9, 16)	0.451
2015-2009	-47.5	(-98.5, 3.4)	0.088
2016-2009	-55.4	(-106.4, -4.5)	0.023
2017-2009	-55.5	(-106.5, -4.6)	0.022
2018-2009	-50.0	(-101, 0.9)	0.058
2019-2009	-42.4	(-93.4, 8.6)	0.188
2011-2010	-4.5	(-55.5, 46.4)	1,000
2012-2010	-22.4	(-73.4, 28.5)	0.928
2013-2010	-24.6	(-75.6, 26.3)	0.873
2014-2010	-31.0	(-81.9, 20)	0.627
2015-2010	-43.6	(-94.6, 7.4)	0.159
2016-2010	-51.5	(-102.5, -0.6)	0.045
2017-2010	-51.6	(-102.5, -0.6)	0.045
2018-2010	-46.1	(-97.1, 4.8)	0.109
2019-2010	-38.5	(-89.4, 12.5)	0.308
2012-2011	-17.9	(-68.8, 33.1)	0.985
2013-2011	-20.1	(-71.1, 30.8)	0.965
2014-2011	-26.5	(-77.4, 24.5)	0.814
2015-2011	-39.1	(-90, 11.9)	0.287
2016-2011	-47.0	(-97.9, 4)	0.096
2017-2011	-47.1	(-98, 3.9)	0.094
2018-2011	-41.6	(-92.6, 9.4)	0.209
2019-2011	-33.9	(-84.9, 17)	0.493
2013-2012	-2.2	(-53.2, 48.7)	1,000
2014-2012	-8.6	(-59.5, 42.4)	1,000
2015-2012	-21.2	(-72.1, 29.8)	0.950
2016-2012	-29.1	(-80.1, 21.9)	0.710
2017-2012	-29.2	(-80.1, 21.8)	0.707
2018-2012	-23.7	(-74.7, 27.2)	0.898
2019-2012	-16.1	(-67, 34.9)	0.994
2014-2013	-6.3	(-57.3, 44.6)	1,000
2015-2013	-19.0	(-69.9, 32)	0.977
2016-2013	-26.9	(-77.8, 24.1)	0.799
2017-2013	-26.9	(-77.9, 24)	0.796
2018-2013	-21.5	(-72.4, 29.5)	0.945
2019-2013	-13.8	(-64.8, 37.1)	0.998
2015-2014	-12.6	(-63.6, 38.3)	0.999
2016-2014	-20.5	(-71.5, 30.4)	0.959
2017-2014	-20.6	(-71.6, 30.3)	0.958
2018-2014	-15.1	(-66.1, 35.8)	0.996

2019-2014	-7.5	(-58.4, 43.5)	1,000
2016-2015	-7.9	(-58.9, 43)	1,000
2017-2015	-8.0	(-58.9, 43)	1,000
2018-2015	-2.5	(-53.5, 48.4)	1,000
2019-2015	5.1	(-45.8, 56.1)	1,000
2017-2016	-0.1	(-51, 50.9)	1,000
2018-2016	5.4	(-45.6, 56.3)	1,000
2019-2016	13.0	(-37.9, 64)	0.999
2018-2017	5.5	(-45.5, 56.4)	1,000
2019-2017	13.1	(-37.8, 64.1)	0.999
2019-2018	7.7	(-43.3, 58.6)	1,000

Table 2. Number in hospitalizations per 100,000 population per region and quadriennium

	GL	SQ	SQM	Value F	P-value
Region	4	1755828.61	438957.15	879.60	<0.001
quadriennium	two	20123.52	10061.76	20.16	<0.001
waste	53	26449.31	499.04		

ANOVA Two-way

We notice, through the p-value, that there is a difference for both Region (p-value= <0.001) as for Quadrennial (p-value= <0.001). The averages of Hospitalizations/100,000 inhab in ascending order of Region were: North (average=319.53, SD=30.25), Northeast (average=439.75, SD=12.89), Midwest (average=517.58, SD=51.52), Southeast (average=587.52, SD=16.06), South (average=831.38, SD=15.5). The test of comparisons tukey multiples pointed out differences between all groups studied, such as we can to see at table a follow:

	Difference	IC.95	P.value
Northeast-Center West	-77.8	(-103.6, -52.1)	<0.001
North-Center West	-198.1	(-223.8, -172.3)	<0.001
Southeast-Center West	69.9	(44.2, 95.7)	<0.001

South-Center West	313.8	(288, 339.6)	<0.001
North, Northeast	-120.2	(-146, -94.5)	<0.001
Southeast-Northeast	147.8	(122, 173.5)	<0.001
south-northeast	391.6	(365.9, 417.4)	<0.001
Southeast-North	268.0	(242.2, 293.7)	<0.001
South North	511.9	(486.1, 537.6)	<0.001
south-southeast	243.9	(218.1, 269.6)	<0.001

The averages of Hospitalizations/100,000hab in ascending order of Quadrennial were: 2016 The 2019 (average=518.86, DP=191.12), 2012 The 2015 (average=535.37, DP=166.31), 2008 to 2011 (average=563.23, SD=172.11). Tukey's multiple comparison testpointed the following differences:

We can check those results in Following table:

	**Difference**	**IC.95**	**P.value**
2012 to 2015-2008 to 2011	-27.9	(-44.9, -10.8)	<0.001
2016 to 2019-2008 to 2011	-44.4	(-61.4, -27.3)	<0.001
2016 to 2019-2012 to 2015	-16.5	(-33.5, 0.5)	0.059

Table3. Number in hospitalizations per age group and quadriennium

	**GL**	**SQ**	**SQM**	**Value F**	**P-value**
Range age	6	557275124683	92879187447	1323.46	<0.001
quadriennium	two	56586554	28293277	0.40	0.67
waste	75	5263422318	70178964		

ANOVA Two-way

We notice, through the p-value that there is a difference for Age Group (p-value= <0.001) butnot for Quadrennial (p-value= 0.67). The averages of Hospitalizations in ascending order in Range Age were: 20 The 29 years old (average=34052, DP=4546.79), 30 The 39 years old

(average=69388.33, DP=5265.2), 40 The 49 years old (average=133405.33, DP=7974.53),

80 years and over (average=150348.25, SD=5974.42), 50 to 59 years (average=224233.08, DP=4188.27), 70 at 79 years old (average=234585.17, DP=6416.83), 60 to 69 years old (mean=268997.75, SD=16650.45). Tukey's multiple comparison test pointed the following differences:
We can check those results in following table:

	Difference	IC.95	P.value
30 to 39 years-20 to 29 years old	35336.3	(24974.3, 45698.3)	<0.001
40 to 49 years-20 to 29 years old	99353.3	(88991.3, 109715.3)	<0.001
50 to 59 years-20 to 29 years old	190181.1	(179819.1, 200543.1)	<0.001
60 to 69 years-20 to 29 years old	234945.7	(224583.8, 245307.7)	<0.001
70 to 79 years-20 to 29 years old	200533.2	(190171.2, 210895.2)	<0.001
80 years old and more-20 to 29 years	116296.2	(105934.3, 126658.2)	<0.001
40 to 49 years-30 to 39 years old	64017.0	(53655, 74379)	<0.001
50 to 59 years-30 to 39 years old	154844.8	(144482.8, 165206.7)	<0.001
60 to 69 years-30 to 39 years old	199609.4	(189247.4, 209971.4)	<0.001
70 to 79 years-30 to 39 years old	165196.8	(154834.8, 175558.8)	<0.001
80 years old and more-30 to 39 years	80959.9	(70597.9, 91321.9)	<0.001
50 to 59 years-40 to 49 years old	90827.8	(80465.8, 101189.7)	<0.001
60 to 69 years-40 to 49 years old	135592.4	(125230.4, 145954.4)	<0.001
70 to 79 years-40 to 49 years old	101179.8	(90817.8, 111541.8)	<0.001
80 years old and more-40 to 49 years	16942.9	(6580.9, 27304.9)	<0.001
60 to 69 years-50 to 59 years old	44764.7	(34402.7, 55126.7)	<0.001
70 to 79 years-50 to 59 years old	10352.1	(-9.9, 20714.1)	0.05
80 years old and more-50 to 59 years	-73884.8	(-84246.8, -63522.8)	<0.001
70 to 79 years-60 to 69 years old	-34412.6	(-44774.6, -24050.6)	<0.001
80 years old and more-60 to 69 years	-118649.5	(-129011.5, -108287.5)	<0.001
80 years old and more-70 at 79 years old	-84236.9	(-94598.9, -73874.9)	<0.001

Table 4. Number in hospitalizations per Age Group and quadriennium

	GL	SQ	SQM	Value F	P-value
Gender	1	46023551	46023551	0.22	0.641
quadrienniu m	two	198052940	99026470	0.48	0.624
waste	20	4104365526	205218276		

ANOVA Two-way

We notice, through the p-value that there is no difference even for Gender (p-value= 0.641)nor for quadriennium (p-value= 0.624).

Table 5. Number in deaths by 100,000 inhabitants by region and year

	GL	SQ	SQM	Value F	P-value
Region	4	6075.40	1518.85	715.37	<0.001
Year	11	173.65	15.79	7.44	<0.001
waste	44	93.42	2.12		

We notice, through the p-value, that there is a difference for both Region (p-value= <0.001) as for Year (p-value= <0.001). The averages of deaths/100,000 inhab in order region were: North (average=25.4, SD=2.06), Northeast (average=36.56, SD=3.17), Midwest (average=39.48, SD=2.12), Southeast (average=49.42, SD=1.45), South (average=54.04, SD=1.85). Tukey's multiple comparison test showed differences in between all groups studied, how can we to see at following table:

	Difference	IC.95	P.value
Northeast-Center West	-2.9	(-4.6, -1.2)	<0.001
North-Center West	-14.1	(-15.8, -12.4)	<0.001
Southeast-Center West	9.9	(8.2, 11.6)	<0.001
South-Center West	14.6	(12.9, 16.3)	<0.001
North, Northeast	-11.2	(-12.9, -9.5)	<0.001
Southeast-Northeast	12.9	(11.2, 14.6)	<0.001
south-northeast	17.5	(15.8, 19.2)	<0.001
Southeast-North	24.0	(22.3, 25.7)	<0.001
South North	28.6	(26.9, 30.3)	<0.001
south-southeast	4.6	(2.9, 6.3)	<0.001

The averages of deaths/100,000 inhab in ascending order of Year were: 2008 (average=37.31, SD=12.8), 2009 (average=39.53, SD=12.22), 2012 (average=39.94, SD=10.29), 2014 (average=39.99, SD=10.93), 2013 (average=40.56, SD=10.93), 2010 (average=40.77, SD=12.42), 2011 (average=40.96, SD=10.97), 2017 (average=41.42, DP=11.08), 2015 (average=41.71, DP=10.95), 2018 (average=42.31, DP=11.29), 2016
(mean=43.2, SD=11), 2019 (mean=44.05, SD=10.89). The test of comparisons multiple in tukey pointed out the following differences. We can check those results in the next table:

	Difference	IC.95	P.value
2009-2008	2.2	(-1, 5.4)	0.423
2010-2008	3.5	(0.3, 6.6)	0.022
2011-2008	3.6	(0.5, 6.8)	0.013
2012-2008	2.6	(-0.5, 5.8)	0.194
2013-2008	3.3	(0.1, 6.4)	0.04
2014-2008	2.7	(-0.5, 5.9)	0.172
2015-2008	4.4	(1.2, 7.6)	0.001
2016-2008	5.9	(2.7, 9.1)	<0.001
2017-2008	4.1	(0.9, 7.3)	0.003
2018-2008	5.0	(1.8, 8.2)	<0.001
2019-2008	6.7	(3.6, 9.9)	<0.001
2010-2009	1.2	(-1.9, 4.4)	0.967
2011-2009	1.4	(-1.7, 4.6)	0.917
2012-2009	0.4	(-2.8, 3.6)	1
2013-2009	1.0	(-2.1, 4.2)	0.992

2014-2009	0.5	(-2.7, 3.6)	1
2015-2009	2.2	(-1, 5.4)	0.446
2016-2009	3.7	(0.5, 6.9)	0.012
2017-2009	1.9	(-1.3, 5.1)	0.655
2018-2009	2.8	(-0.4, 6)	0.136
2019-2009	4.5	(1.3, 7.7)	<0.001
2011-2010	0.2	(-3, 3.4)	1
2012-2010	-0.8	(-4, 2.3)	0.999
2013-2010	-0.2	(-3.4, 3)	1
2014-2010	-0.8	(-4, 2.4)	0.999
2015-2010	0.9	(-2.2, 4.1)	0.996
2016-2010	2.4	(-0.7, 5.6)	0.291
2017-2010	0.6	(-2.5, 3.8)	1
2018-2010	1.5	(-1.6, 4.7)	0.871
2019-2010	3.3	(0.1, 6.5)	0.038
2012-2011	-1.0	(-4.2, 2.2)	0.993
2013-2011	-0.4	(-3.6, 2.8)	1
2014-2011	-1.0	(-4.1, 2.2)	0.995
2015-2011	0.8	(-2.4, 3.9)	0.999
2016-2011	2.2	(-0.9, 5.4)	0.403
2017-2011	0.5	(-2.7, 3.6)	1
2018-2011	1.4	(-1.8, 4.5)	0.94
2019-2011	3.1	(-0.1, 6.3)	0.063
2013-2012	0.6	(-2.6, 3.8)	1
2014-2012	0.1	(-3.1, 3.2)	1
2015-2012	1.8	(-1.4, 5)	0.739
2016-2012	3.3	(0.1, 6.4)	0.039
2017-2012	1.5	(-1.7, 4.7)	0.897
2018-2012	2.4	(-0.8, 5.6)	0.323
2019-2012	4.1	(0.9, 7.3)	0.003
2014-2013	-0.6	(-3.8, 2.6)	1
2015-2013	1.1	(-two, 4.3)	0.982
2016-2013	2.6	(-0.5, 5.8)	0.19
2017-2013	0.9	(-2.3, 4)	0.998
2018-2013	1.8	(-1.4, 4.9)	0.753
2019-2013	3.5	(0.3, 6.7)	0.021
2015-2014	1.7	(-1.5, 4.9)	0.773
2016-2014	3.2	(0, 6.4)	0.046
2017-2014	1.4	(-1.8, 4.6)	0.917
2018-2014	2.3	(-0.9, 5.5)	0.355
2019-2014	4.1	(0.9, 7.2)	0.003
2016-2015	1.5	(-1.7, 4.7)	0.893
2017-2015	-0.3	(-3.5, 2.9)	1
2018-2015	0.6	(-2.6, 3.8)	1
2019-2015	2.3	(-0.8, 5.5)	0.346
2017-2016	-1.8	(-5, 1.4)	0.732

2018-2016	-0.9	(-4.1, 2.3)	0.998
2019-2016	0.8	(-2.3, 4)	0.999
2018-2017	0.9	(-2.3, 4.1)	0.998
2019-2017	2.6	(-0.6, 5.8)	0.194
2019-2018	1.7	(-1.4, 4.9)	0.764

Table 6. Number in Deaths per 100,000 population per region and quadriennium

	GL	SQ	SQM	Value F	P-value
Region	4	6075.40	1518.85	487.47	<0.001
quadriennium	two	101.94	50.97	16.36	<0.001
waste	53	165.14	3.12		

We notice, through the p-value, that there is a difference for both Region (p-value= <0.001) as for Quadrennial (p-value= <0.001). The averages of deaths/100,000 inhab in orderregion were: North (average=25.4, SD=2.06), Northeast (average=36.56, SD=3.17), Midwest (average=39.48, SD=2.12), Southeast (average=49.42, SD=1.45), South (average=54.04, SD=1.85). Tukey's multiple comparison test showed differences in between all groups studied, how we can see at table:

	Difference	IC.95	P.value
Northeast-Center West	-2.9	(-5, -0.9)	0.002
North-Center West	-14.1	(-16.1, -12)	<0.001
Southeast-Center West	9.9	(7.9, 12)	<0.001
South-Center West	14.6	(12.5, 16.6)	<0.001
North, Northeast	-11.2	(-13.2, -9.1)	<0.001
Southeast-Northeast	12.9	(10.8, 14.9)	<0.001
south-northeast	17.5	(15.4, 19.5)	<0.001
Southeast-North	24.0	(22, 26.1)	<0.001
South North	28.6	(26.6, 30.7)	<0.001
south-southeast	4.6	(2.6, 6.7)	<0.001

The averages of deaths/100,000 inhab in ascending order of Quadrennial were: 2008 to2011 (average=39.64, DP=11.22), 2012 The 2015 (average=40.55, DP=9.92), 2016 to 2019 (mean=42.75, SD=10.2). The tukey multiple comparison test pointed out the following differences. We can check those results at following table:

	Difference	IC.95	P.value
2012 to 2015-2008 to 2011	0.9	(-0.4, 2.3)	0.242
2016 to 2019-2008 to 2011	3.1	(1.8, 4.5)	<0.001
2016 to 2019-2012 to 2015	2.2	(0.8, 3.5)	<0.001

ANOVA Two-way

Table 7. Number in deaths by range age and quadriennium

	GL	SQ	SQM	Value F	P-value
Range age	6	7045372005	1174228667	958.77	<0.001
quadriennium	two	36727564	18363782	14.99	<0.001
Residuals	75	91854330	1224724		

We notice, through the p-value that there is a difference for Age Group (p-value= <0.001) as for Quadrennial (p-value= <0.001). The averages of deaths in ascending order of Range age were: 20 The 29 years old (average=1023.75, DP=89.28), 30 The 39 years old (mean=2271.83, SD=102.5), 40 to 49 years old (mean=5704, SD=297.86), 50 to 59 years old (average=12127.08, DP=378.88), 60 The 69 years old (average=19536.92, DP=1734.32), 80 years oldand more (average=23638.58, DP=2437.26), 70 The 79 years old (average=23943.92, DP=1578.11).
We can check Those results in following table:

	Difference	95%CI	P-value
30 to 39 years-20 to 29 years old	1248.1	(-120.8, 2616.9)	0.097
40 to 49 years-20 to 29 years old	4680.2	(3311.4, 6049.1)	<0.001
50 to 59 years-20 to 29 years old	11103.3	(9734.5, 12472.2)	<0.001
60 to 69 years-20 to 29 years old	18513.2	(17144.3, 19882)	<0.001
70 to 79 years-20 to 29 years old	22920.2	(21551.3, 24289)	<0.001
80 years old and more-20 to 29 years	22614.8	(21246, 23983.7)	<0.001
40 to 49 years-30 to 39 years old	3432.2	(2063.3, 4801)	<0.001
50 to 59 years-30 to 39 years old	9855.3	(8486.4, 11224.1)	<0.001
60 to 69 years-30 to 39 years old	17265.1	(15896.2, 18633.9)	<0.001
70 to 79 years-30 to 39 years old	21672.1	(20303.2, 23040.9)	<0.001
80 years old and more-30 to 39 years	21366.8	(19997.9, 22735.6)	<0.001
50 to 59 years-40 to 49 years old	6423.1	(5054.2, 7791.9)	<0.001
60 to 69 years-40 to 49 years old	13832.9	(12464.1, 15201.8)	<0.001
70 to 79 years-40 to 49 years old	18239.9	(16871.1, 19608.8)	<0.001
80 years old and more-40 to 49	17934.6	(16565.7, 19303.4)	<0.001

years			
60 to 69 years-50 to 59 years old	7409.8	(6041, 8778.7)	<0.001
70 to 79 years-50 to 59 years old	11816.8	(10448, 13185.7)	<0.001
80 years old and more-50 to 59 years	11511.5	(10142.6, 12880.4)	<0.001
70 to 79 years-60 to 69 years old	4407.0	(3038.1, 5775.9)	<0.001
80 years old and more-60 to 69 years	4101.7	(2732.8, 5470.5)	<0.001
80 years old and more-70 to 79 years	-305.3	(-1674.2, 1063.5)	0.994

ANOVA Two-way

At averages in Deaths in order growing in quadriennium were: 2008 The 2011 (average=11875.11, SD=8425.81), 2012 The 2015 (average=12467.79, SD=9308.51), 2016 The 2019 (average=13476.86, DP=10329.52). THE test in comparisons multiple in tukeypointed the following differences. We can check Those results at following table:

	**Difference**	**95%CI**	**P-value**
2012 to 2015-2008 to 2011	592.7	(-114.5, 1299.9)	0.118
2016 to 2019-2008 to 2011	1601.8	(894.5, 2309)	<0.001
2016 to 2019-2012 to 2015	1009.1	(301.9, 1716.3)	0.003

Table 8. Number in deaths by Gender and quadriennium

	GL	SQ	SQM	Value F	P-value
Gender	1	27266148	27266148	10.96	0.003
quadrienniu m	two	128546473	64273236	25.83	<0.001
Residuals	20	49758088	2487904		

We notice, through the p-value that there is a difference for Gender (p-value= 0.003) as for Quadrennial (p-value= <0.001). The averages of deaths in ascending order of Gender were: Female (average=43057.17, SD=2728.34), Male (mean=45188.92, SD=2960.69). Tukey's multiple comparison test showeddifferences in between all you groups studied, how can we to see at table The follow:

Difference	IC.95	P.value
2131.8	(788.5, 3475)	0.003

The averages of deaths in ascending order of Quadrennial were: 2008 to 2011 (average=41562.88, DP=2424.87), 2012 The 2015 (average=43637.25, DP=1492.82), 2016 The 2019 (average=47169, SD=1701.46). Tukey's multiple comparison test pointed out differences between all the groups studied, as we can see in the tablefollow:

	Difference	IC.95	P.value
2012 to 2015-2008 to 2011	2074.4	(79.1, 4069.7)	0.041
2016 to 2019-2008 to 2011	5606.1	(3610.8, 7601.4)	<0.001
2016 to 2019-2012 to 2015	3531.8	(1536.5, 5527)	<0.001

Table 9. Rate in mortality by Region and Year

	GL	SQ	SQM	Value F	P-value
Region	4	29.70	7.42	26.68	<0.001
Year	11	26.62	2.42	8.69	<0.001
Residuals	44	12.24	0.28		

We notice, through the p-value, that there is a difference for both Region (p-value= <0.001) as for Year (p-value= <0.001). The averages of Mortality Rate in order region were: South (average=6.5, SD=0.2), Midwest (average=7.67, SD=1.02), North (average=8.13, SD=1.31), Northeast (average=8.32, SD=0.8), Southeast(mean=8.41, SD=0.26). The tukey multiple comparison test pointed out the following differences: We can check those results in following table:

	Difference	IC.95	P.value
Northeast-Center West	0.7	(0, 1.3)	0.032
North-Center West	0.5	(-0.2, 1.1)	0.22
Southeast-Center West	0.7	(0.1, 1.4)	0.011
South-Center West	-1.2	(-1.8, -0.6)	<0.001
North, Northeast	-0.2	(-0.8, 0.4)	0.901
Southeast-Northeast	0.1	(-0.5, 0.7)	0.994
south-northeast	-1.8	(-2.4, -1.2)	<0.001
Southeast-North	0.3	(-0.3, 0.9)	0.692
South North	-1.6	(-2.2, -1)	<0.001
south-southeast	-1.9	(-2.5, -1.3)	<0.001

The averages of Mortality Rate in ascending order of Year were: 2008 (average=6.72, SD=0.81), 2009 (average=6.99, SD=0.79), 2010 (average=7.28, SD=0.78), 2011 (average=7.4, DP=0.8), 2012 (average=7.48, DP=0.82), 2013 (average=7.58, DP=0.7), 2014 (average=7.6, SD=0.66), 2015 (average=8.2, DP=0.86), 2017 (average=8.41, SD=1.15), 2018 (average=8.48, DP=1.06), 2016 (average=8.77, DP=1.18), 2019 (mean=8.78, SD=1.33). The tukey multiple comparison test pointed out the following differences:
We can check these results in the table:

	Difference	IC.95	P.value
2009-2008	0.3	(-0.9, 1.4)	1
2010-2008	0.6	(-0.6, 1.7)	0.866
2011-2008	0.7	(-0.5, 1.8)	0.662
2012-2008	0.8	(-0.4, 1.9)	0.502
2013-2008	0.9	(-0.3, two)	0.329
2014-2008	0.9	(-0.3, two)	0.291
2015-2008	1.5	(0.3, 2.6)	0.003
2016-2008	2.0	(0.9, 3.2)	<0.001
2017-2008	1.7	(0.5, 2.8)	<0.001
2018-2008	1.8	(0.6, 2.9)	<0.001
2019-2008	2.1	(0.9, 3.2)	<0.001
2010-2009	0.3	(-0.9, 1.4)	0.999
2011-2009	0.4	(-0.7, 1.6)	0.981
2012-2009	0.5	(-0.7, 1.6)	0.935
2013-2009	0.6	(-0.6, 1.7)	0.823
2014-2009	0.6	(-0.5, 1.8)	0.784
2015-2009	1.2	(0.1, 2.4)	0.031
2016-2009	1.8	(0.6, 2.9)	<0.001
2017-2009	1.4	(0.3, 2.6)	0.005
2018-2009	1.5	(0.3, 2.6)	0.003
2019-2009	1.8	(0.6, 2.9)	<0.001
2011-2010	0.1	(-1, 1.3)	1
2012-2010	0.2	(-1, 1.4)	1
2013-2010	0.3	(-0.9, 1.4)	0.999
2014-2010	0.3	(-0.8, 1.5)	0.998
2015-2010	0.9	(-0.2, 2.1)	0.239
2016-2010	1.5	(0.3, 2.6)	0.003
2017-2010	1.1	(0, 2.3)	0.059
2018-2010	1.2	(0, 2.3)	0.036
2019-2010	1.5	(0.3, 2.6)	0.003
2012-2011	0.1	(-1.1, 1.2)	1

2013-2011	0.2	(-1, 1.3)	1
2014-2011	0.2	(-1, 1.3)	1
2015-2011	0.8	(-0.4, 1.9)	0.436
2016-2011	1.4	(0.2, 2.5)	0.009
2017-2011	1.0	(-0.1, 2.2)	0.137
2018-2011	1.1	(-0.1, 2.2)	0.087
2019-2011	1.4	(0.2, 2.5)	0.008
2013-2012	0.1	(-1.1, 1.2)	1
2014-2012	0.1	(-1, 1.3)	1
2015-2012	0.7	(-0.4, 1.9)	0.595
2016-2012	1.3	(0.1, 2.4)	0.017
2017-2012	0.9	(-0.2, 2.1)	0.223
2018-2012	1.0	(-0.2, 2.1)	0.149
2019-2012	1.3	(0.1, 2.4)	0.016
2014-2013	0.0	(-1.1, 1.2)	1
2015-2013	0.6	(-0.5, 1.8)	0.774
2016-2013	1.2	(0, 2.3)	0.036
2017-2013	0.8	(-0.3, two)	0.367
2018-2013	0.9	(-0.3, 2.1)	0.261
2019-2013	1.2	(0, 2.3)	0.035
2015-2014	0.6	(-0.6, 1.7)	0.813
2016-2014	1.2	(0, 2.3)	0.044
2017-2014	0.8	(-0.3, two)	0.41
2018-2014	0.9	(-0.3, two)	0.297
2019-2014	1.2	(0, 2.3)	0.042
2016-2015	0.6	(-0.6, 1.7)	0.855
2017-2015	0.2	(-0.9, 1.4)	1
2018-2015	0.3	(-0.9, 1.4)	0.999
2019-2015	0.6	(-0.6, 1.7)	0.846
2017-2016	-0.4	(-1.5, 0.8)	0.994
2018-2016	-0.3	(-1.4, 0.9)	0.999
2019-2016	0.0	(-1.1, 1.2)	1
2018-2017	0.1	(-1.1, 1.2)	1
2019-2017	0.4	(-0.8, 1.5)	0.994
2019-2018	0.3	(-0.9, 1.4)	0.999

Table 10. Rate in Mortality by Region and quadriennium

	GL	SQ	SQM	Value F	P-value
Region	4	29.70	7.42	24.90	<0.001
quadriennium	two	05.23	11.53	38.66	<0.001
Residuals	53	15.80	0.30		

We notice, through the p-value, that there is a difference for both Region (p-value= <0.001) as for Quadrennial (p-value= <0.001). The averages of Mortality Rate in order region were: South (average=6.5, SD=0.2), Midwest (average=7.67, SD=1.02), North (average=8.13, SD=1.31), Northeast (average=8.32, SD=0.8), Southeast (mean=8.41, SD=0.26). The tukey multiple comparison test pointed out the following differences:
We can check those results in Following table:

	Difference	IC.95	P.value
Northeast-Center West	0.7	(0, 1.3)	0.039
North-Center West	0.5	(-0.2, 1.1)	0.248
Southeast-Center West	0.7	(0.1, 1.4)	0.013
South-Center West	-1.2	(-1.8, -0.5)	<0.001
North, Northeast	-0.2	(-0.8, 0.4)	0.911
Southeast-Northeast	0.1	(-0.5, 0.7)	0.994
south-northeast	-1.8	(-2.5, -1.2)	<0.001
Southeast-North	0.3	(-0.3, 0.9)	0.719
South North	-1.6	(-2.3, -1)	<0.001
south-southeast	-1.9	(-2.5, -1.3)	<0.001

The averages of Mortality Rate in ascending Quadrennial order were: 2008 to2011 (average=7.1, DP=0.78), 2012 The 2015 (average=7.72, DP=0.76), 2016 The 2019 (mean=8.61, SD=1.1). The tukey multiple comparison test showed differencesin between all groups studied, how can we to see at table a follow:

	Difference	IC.95	P.value
2012 to 2015-2008 to 2011	0.6	(0.2, 1)	0.002
2016 to 2019-2008 to 2011	1.5	(1.1, 1.9)	<0.001
2016 to 2019-2012 to 2015	0.9	(0.5, 1.3)	<0.001

ANOVA Two-way

Table 11. Rate in Mortality per Range age and quadriennium

	GL	SQ	SQM	Value F	P-value
Region	6	1503.42	250.57	1745.03	<0.001
quadrienniu m	two	6.83	3.42	23.79	<0.001
waste	75	10.77	0.14		

We notice, through the p-value, that there is a difference for both Region (p-value= <0.001) as for Quadrennial (p-value= <0.001). The averages of Mortality Rate in order Region were: 20 to 29 years (mean=3.03, SD=0.22), 30 to 39 years (average=3.29, DP=0.19), 40 at 49 years old (average=4.28, DP=0.15), 50 to 59 years old (mean=5.41, SD=0.17), 60 to 69 years old (mean=7.25, SD=0.24), 70 to 79 years old (mean=10.2, SD=0.48), 80 years and over (mean=15.69, SD=1.09).

We can check those results in following table:

	Difference	IC.95	P.value
30 to 39 years-20 to 29 years old	0.3	(-0.2, 0.7)	0.641
40 to 49 years-20 to 29 years old	1.3	(0.8, 1.7)	<0.001
50 to 59 years-20 to 29 years old	2.4	(1.9, 2.9)	<0.001
60 to 69 years-20 to 29 years old	4.2	(3.8, 4.7)	<0.001
70 to 79 years-20 to 29 years old	7.2	(6.7, 7.6)	<0.001
80 years old and more-20 to 29 years	12.7	(12.2, 13.1)	<0.001
40 to 49 years-30 to 39 years old	1.0	(0.5, 1.5)	<0.001
50 to 59 years-30 to 39 years old	2.1	(1.7, 2.6)	<0.001
60 to 69 years-30 to 39 years old	4.0	(3.5, 4.4)	<0.001
70 to 79 years-30 to 39 years old	6.9	(6.4, 7.4)	<0.001
80 years old and more-30 to 39 years	12.4	(11.9, 12.9)	<0.001
50 to 59 years-40 to 49 years old	1.1	(0.7, 1.6)	<0.001
60 to 69 years-40 to 49 years old	3.0	(2.5, 3.4)	<0.001
70 to 79 years-40 to 49 years old	5.9	(5.5, 6.4)	<0.001
80 years old and more-40 to 49 years	11.4	(10.9, 11.9)	<0.001
60 to 69 years-50 to 59 years old	1.8	(1.4, 2.3)	<0.001
70 to 79 years-50 to 59 years old	4.8	(4.3, 5.3)	<0.001
80 years old and more-50 to 59 years	10.3	(9.8, 10.7)	<0.001
70 to 79 years-60 to 69 years old	2.9	(2.5, 3.4)	<0.001
80 years old and more-60 to 69 years	8.4	(8, 8.9)	<0.001
80 years old and more-70 to 79 years	5.5	(5, 6)	<0.001

The averages of Mortality Rate in ascending Quadrennial order were: 2008 to 2011 (average=6.7, DP=3.94), 2012 The 2015 (average=6.96, DP=4.39), 2016 The 2019 (mean=7.4, SD=4.61). The tukey multiple comparison test showed differences in between all groups studied, how can we to see at table a follow:

	**Difference**	**IC.95**	**P.value**
2012 to 2015-2008 to 2011	0.3	(0, 0.5)	0.037
2016 to2019-2008 to 2011	0.7	(0.4, 0.9)	<0.001
2016 to 2019-2012 to 2015	0.4	(0.2, 0.7)	<0.001

Table 12. Rate in mortality by gender and quadriennium

	GL	SQ	SQM	Value F	P-value
Gender	1	0.67	0.67	12.02	0.002
quadriennium	two	3.43	1.71	30.91	<0.001
Residuals	20	1.11	0.06		

We note, through of p-value what there is difference so much for Gender (p-value= 0.002) as for Quadrennial (p-value= <0.001). The averages of Mortality Rate in ordergrowing in Gender were: Female (average=7.75, DP=0.56), Male (mean=8.08, SD=0.32). THE test of multiple comparisons in tukey pointed differences in between all groups studied, how can we to see at following table:

Difference	IC.95	P.value
0.3	(0.1, 0.5)	0.002

The averages of Mortality Rate in ascending Quadrennial order were: 2008 to 2011 (average=7.48, DP=0.39), 2012 to 2015 (average=7.86, DP=0.3), 2016 to 2019 (mean=8.4, SD=0.12). The tukey multiple comparison test showed differencesin between all groups studied, how can we to see at table a follow:

	Difference	IC.95	P.value
2012 to 2015-2008 to 2011	0.4	(0.1, 0.7)	0.011
2016 to 2019-2008 to 2011	0.9	(0.6, 1.2)	<0.001
2016 to 2019-2012 to 2015	0.5	(0.2, 0.8)	<0.001
