# Supplementary material for: The impact of the strategic action plan to combat chronic non-communicable diseases on hospital admissions and deaths from cardiovascular diseases in Brazil
Source: PLoS One. 2022 Jun 8;17(6):e0269583. doi: 10.1371/journal.pone.0269583 (PMC9176809; doi:10.1371/journal.pone.0269583)
Supplement: Attachment — Submitted filename: Reviewer comments_Win Thuzar Aye.docx [file pone.0269583.s002.docx]

This paper is interesting study on the evaluation of the impact of the strategic action plan to combat chronic non-communicable diseases on hospital admissions and deaths from cardiovascular diseases in Brazil. The study further analysed time trend from 2008 to 2019 and useful in supporting health promotion public policies. I have provided a few suggestions to improve the manuscript below:

In the abstract, Please express HF.

In data extraction, how do you check the data validity and accuracy?

Line 115 - please describe ‘age group (20 to 80 years or older)’.

Line 116 – what does it mean, “The number of hospital admissions and deaths per 100,000 inhabitants was calculated by the ratio between the number of hospital admissions and deaths recorded and the estimate of the Brazilian population, multiplied by 100,000, according to the population projections made by the IBGE.” Please provide clear information about mortality rate.

Line 133- I didn’t find these data in Table 1 “Heart Failure (20.68%), other ischemic heart diseases (13.54%) and stroke (11.98%), Table 1”. Please check it.

Line 142 – What do you mean this sentence “When evaluating the difference between region of residence and year in relation to hospital admissions/100 thousand inhabitants, it is possible to observe that there is a difference for both region (p<0.001)”

Figure 1, 5 and 9 should be describe with Line graph.

In discussion, there is a lot of repetition of the results. It is possible to have a good discussion without putting so many results from the study itself. It is also possible to dialogue with the studies that served as a basis without putting their numerical results. The results of incidence and regional distribution as well as the time trend should compare with other countries.

According to your objective, you should firstly discuss with line 392-

“The main findings in the study with regard to hospital admissions for CVDs after implementing the Strategic Action Plan for Coping with NCDs (2011-2022) were: 1) a 25.77%----------”. Please provide more information why decrease and increase on hospital admissions, deaths and mortality rate.
