# Supplementary material for: The impact of the strategic action plan to combat chronic non-communicable diseases on hospital admissions and deaths from cardiovascular diseases in Brazil
Source: PLoS One. 2022 Jun 8;17(6):e0269583. doi: 10.1371/journal.pone.0269583 (PMC9176809; doi:10.1371/journal.pone.0269583)
Supplement: Attachment — Submitted filename: Response to reviewers.docx [file pone.0269583.s003.docx]

PONE-D-22-03798 – “Response to reviewers”

The impact of the strategic action plan to combat chronic non-communicable diseases on hospital admissions and deaths from cardiovascular diseases in Brazil

PLOS ONE

Thank you for taking the time to review our article. We revised the manuscript in accordance with the reviewers' comments and suggestions. Attached is the new manuscript along with our point-by-point responses.

Reviewer #1: General comment on the article:

1. The article showed an important subject which is the impact of strategic planning in the disease prevention and control.

In the introduction the authors did not give the details about the strategy such as the goals, the objectives, the indicators that was expected, some activities for the implementation, so the readers know exactly how the strategy was structured, and the result expected from it. This is important if there will be any amendments that should be done.

Author response: Thank you so much for your comment. The suggestion was accepted and so we provided this information in paragraph 7, lines 90-96.

2. The article have big data that give real insights on NCDs in the country, the article showed result with stratification by years, age, region , gender and grouping by years and age, the result showed too much details that does not needed to be present in the context but can be shown in tables and graphs, Too much numbers and percentages in the context may make the readers lost, we recommend less numbers and keep the important one and explain if there is trend.

Author response: Thank you for your comment.

3. The discussion was better than the result in the way it was organized and really showed what the author wants to answer, it though needed to be compared with more articles in the region and the world, and the author may give some reasons for why the result was like that (professional opinion based on author observation or other studies in the country)

Author response: We really appreciate your suggestion. This information was added in paragraph 16 of discussion, lines 434-443.

4. One limitation of the study is it does not consider any changes in risk factors for NCDs as they change before the changes happened in the hospitalization and the mortality rate, but this may be not available in the data used by the authors.

Author response: All these data were extracted from a platform implemented by the Brazilian government for the publication of this king of data, called DATASUS, available on the website [www.datasus.gov.br](http://www.datasus.gov.br). DATASUS is the service department of the Brazilian Unified Health System (SUS), with the objective of norms, guidelines, and procedures for downloading information and contracting informatics services within agencies and entities of the Brazilian Ministry of Health. It is responsible to ensure SUS managers and the Ministry to access technology systems information and databases. However, there are so many information gaps, including these changes in risk factors and their changes over the analyzed years, there were not available for analyzes.

5. In the last section there should be recommendations to improve the strategy and recommendations for further studies

Author response: Thank you so much for your comment. The suggestion was accepted and so we provided this information in the last paragraph, lines 464-470.

Reviewer #2: General comment on the article:

1. This is an interesting manuscript which highlighted the cardiovascular risk in hospital admission and death. Samples based on secondary data is adequate and statistical analysis is suitable to draw conclusion about the study. Authors tried to explore the data as much as possible but descriptive statistics of hospital admission, death, and mortality rate; and the results by two-way ANOVA test should be added by the tables as supporting information to be more clear interpretation.

Author response: Thank you for your comment. We added a file named “Supporting Information” with all the results by two-way ANOVA, with post-hocs.

Reviewer #3: General comment on the article:

1. This paper is interesting study on the evaluation of the impact of the strategic action plan to combat chronic non-communicable diseases on hospital admissions and deaths from cardiovascular diseases in Brazil. The study further analyzed time trend from 2008 to 2019 and useful in supporting health promotion public policies. I have provided a few suggestions to improve the manuscript below:

In the abstract, Please express HF.

Author response: Thank you so much for your comment. However, this acronym does not appear in this section.

2. In data extraction, how do you check the data validity and accuracy?

Author response: The data validity and accuracy were not assessed by statistical tests since the extraction was performed by only one author. Then, the information was checked and verified by a second author.

3. Line 115 - please describe ‘age group (20 to 80 years or older)’.

Author response: Thank you so much for your comment. The suggestion was accepted, and the description was made.

4. Line 116 – what does it mean, “The number of hospital admissions and deaths per 100,000 inhabitants was calculated by the ratio between the number of hospital admissions and deaths recorded and the estimate of the Brazilian population, multiplied by 100,000, according to the population projections made by the IBGE.” Please provide clear information about mortality rate.

Author response: Thank you for your comment. Since the values presented in the DATASUS extraction were raw values of hospital admissions and deaths, we carefully considered the population of each Brazilian region so that the data were proportional. Thus, we improve the sentence to “The number of hospital admissions and deaths was calculated by the ratio between the number of hospital admissions and deaths recorded and the estimate of each region Brazilian population, according to the IBGE.”

5. Line 133- I didn’t find these data in Table 1 “Heart Failure (20.68%), other ischemic heart diseases (13.54%) and stroke (11.98%), Table 1”. Please check it.

Author response: Thank you so much for your comment. The sentence was revised.

6. Line 142 – What do you mean this sentence “When evaluating the difference between region of residence and year in relation to hospital admissions/100 thousand inhabitants, it is possible to observe that there is a difference for both region (p<0.001)”

Author response: Thank you so much for your comment. The sentence was revised.

7. Figure 1, 5 and 9 should be describe with Line graph.

Author response: The suggestion was accepted.

8. In discussion, there is a lot of repetition of the results. It is possible to have a good discussion without putting so many results from the study itself. It is also possible to dialogue with the studies that served as a basis without putting their numerical results. The results of incidence and regional distribution as well as the time trend should compare with other countries.

Author response: The suggestion was very important. We added this information according to Heart Disease and Stroke Statistics, 2021 Update, A Report from the American Heart Association and European Cardiovascular Disease Statistics 2017 edition.

9.According to your objective, you should firstly discuss with line 392 - “The main findings in the study with regard to hospital admissions for CVDs after implementing the Strategic Action Plan for Coping with NCDs (2011-2022) were: 1) a 25.77%----------”. Please provide more information why decrease and increase on hospital admissions, deaths and mortality rate.

Author response: The suggestion was accepted and so we reformed the information in lines 425-419.
